# Accuracy of Continuous Glucose Monitoring Measurements in Normo-Glycemic Individuals

**DOI:** 10.1371/journal.pone.0139973

**Published:** 2015-10-07

**Authors:** Abimbola A. Akintola, Raymond Noordam, Steffy W. Jansen, Anton J. de Craen, Bart E. Ballieux, Christa M. Cobbaert, Simon P. Mooijaart, Hanno Pijl, Rudi G. Westendorp, Diana van Heemst

**Affiliations:** 1 Department of Gerontology and Geriatrics, Leiden University Medical Center, Leiden, the Netherlands; 2 Department of Clinical Chemistry, Leiden University Medical Center, Leiden, The Netherlands; 3 Department of Endocrinology, Leiden University Medical Center, Leiden, the Netherlands; 4 Department of Public Health, University of Copenhagen, Copenhagen, Denmark; University of Catanzaro Magna Graecia, ITALY

## Abstract

**Background:**

The validity of continuous glucose monitoring (CGM) is well established in diabetic patients. CGM is also increasingly used for research purposes in normo-glycemic individuals, but the CGM validity in such individuals is unknown. We studied the accuracy of CGM measurements in normo-glycemic individuals by comparing CGM-derived versus venous blood-derived glucose levels and measures of glycemia and glycemic variability.

**Methods:**

In 34 healthy participants (mean age 65.7 years), glucose was simultaneously measured every 10 minutes, via both an Enlite^®^ CGM sensor, and in venous blood sampled over a 24-hour period. Validity of CGM-derived individual glucose measurements, calculated measures of glycemia over daytime (09:00h-23:00h) and nighttime (23:00h-09:00h), and calculated measures of glycemic variability (e.g. 24h standard deviation [SD]) were assessed by Pearson correlation coefficients, mean absolute relative difference (MARD) and paired t-tests.

**Results:**

The median correlation coefficient between CGM and venous glucose measurements per participant was 0.68 (interquartile range: 0.40–0.78), and the MARD was 17.6% (SD = 17%). Compared with venous sampling, the calculated measure of glycemia during daytime was 0.22 mmol/L higher when derived from CGM, but no difference was observed during nighttime. Most measures of glycemic variability were lower with CGM than with venous blood sampling (e.g., 24h SD: 1.07 with CGM and 1.26 with venous blood; p-value = 0.004).

**Conclusion:**

In normo-glycemic individuals, CGM-derived glucose measurements had good agreement with venous glucose levels. However, the measure of glycemia was higher during the day and most measures of glycemic variability were lower when derived from CGM.

## Introduction

Continuous glucose monitoring (CGM) is a minimally invasive method that has been approved for ambulant glucose monitoring in patients with diabetes mellitus [1]. For the purpose of patient care, the validity of different CGM devices has been studied against glucose measures obtained with another CGM device [2], glucometers [3, 4], capillary blood [5, 6] and venous blood taken at random time points [7]. Recently, studies have also been conducted in which CGM glucose measurements were compared with frequently sampled venous blood glucose measurements [8–11]. In general, these studies have shown that glucose measurements derived with a CGM device were comparable to venous blood glucose measurements [8–11]. For example, the CGM Enlite^®^ provides accurate glucose readings (correct detection of existing or predicted hypo- and hyperglycemia) for up to six consecutive days in diabetic patients when calibrated against capillary glucose three to four times a day [8].

In addition to patient care, CGM has also been increasingly used in epidemiological studies in healthy volunteers [12–14]. For research, the main advantage of CGM is that the device is portable, easy to use, cost effective, and can be used during normal daily activities. After processing, the device provides information on 24-hour glucose rhythms for up to six consecutive days. From this data, measures of glycemia and glycemic variability can be calculated. However, to date, the validity of the estimates for glycemia and glycemic variability as well as the glucose levels themselves have not been studied in normo-glycemic individuals.

In the present study, we conducted a validation study of glucose measurements obtained with CGM using the Enlite^®^ sensor in normo-glycemic individuals. For this, we studied accuracy of CGM measurements by comparing CGM-derived glucose levels and measures of glycemia and glycemic variability with those obtained from simultaneously sampled venous blood.

## Materials and Methods

### Ethics statement

The Medical Ethical Committee of Leiden University Medical Center approved this study, and all investigations have been conducted according to the principles expressed in the Declaration of Helsinki. Written informed consent was obtained from all study participants.

### Study participants

The present study was embedded in the Switchbox Study [15], which was a sub-study of the Leiden Longevity Study (LLS). The LLS was originally designed to investigate genetic and phenotypic biomarkers associated with human longevity. In total, the LLS comprised 2,415 participants (1,671 offspring from nonagenarian siblings and 744 partners thereof). A more detailed description of the study design and recruitment strategy has been described elsewhere [16].

Of these, a subsample of 38 non-diabetic participants underwent 24-hour venous blood sampling. To be included, the participants had to have a fasting glucose level <7 mmol/L, hemoglobin >7.1 mmol/L, a body mass index (BMI) between 19 kg/m^2^ and 33 kg/m^2^ and be free of any significant chronic disease. Exclusion criteria that were considered for participation in the 24-hour venous blood sampling included, among others, use of any medication known to influence lipolysis, thyroid function, glucose metabolism, growth hormone secretion or any other hormonal axis, difficulties in inserting and maintaining an intravenous catheter, blood donation within the last two months, smoking and alcohol addiction, and extreme diet therapies, as has been described in more detail elsewhere [17]. Of the 38 participants, 34 had simultaneously measured glucose levels from CGM and venous blood (no CGM data could be uploaded for four participants).

### Study and sampling procedure

After an overnight fast of 10–14 hours, a catheter, for the purpose of venous blood sampling, was inserted in the non-dominant hand before the start of the study. Blood sampling started at 09.00h and continued for 24 hours. During this period, 2 ml of blood were collected every 10 minutes in a serum separator (SST)-tube.

During the study period, participants received three standardized meals at three fixed time points (namely, between 09.00h-10.00h, 12.00h-13.00h and 18.00h-19.00h). All meals consisted of 600 kcal Nutridrink (Nutricia Advanced Medical Nutrition, Zoetermeer, the Netherlands). All participants were sampled in the same room with standardized ambient conditions. Participants were not allowed to sleep during the day; lights were turned off between 23.00h to 08.00h to allow the participants to sleep.

#### Continuous glucose monitoring

Each participant was assigned to a *i*Pro^®^ 2 MiniMed^®^ continuous glucose monitoring System (Medtronic MiniMed Inc., Northridge, CA, USA). The system comprised of (i) a sterile, single use electrode sensor system (ENLiTE^®^; Medtronic MiniMed Inc., released 2010), inserted into the interstitial fluid, that continuously generates an electrical current proportional to the glucose concentration, (ii) a Sen-serter for sensor insertion, (iii) a *i*Pro2 recorder that digitally stores the average sensor current every 5 minutes, and (iv) a dock wired to a personal computer (PC) with CareLink^®^ iPro, through which data from iPro2 is uploaded through dock to PC with Internet connection to CareLink iPro. The glucose sensor was inserted into the subcutaneous abdominal fat tissue the day before the study, to allow for sensor equilibration and for resolution of any insertion-induced micro-hematoma. This procedure has been validated previously and provides accurate CGM glucose recordings over a longer period [8].

The CGM glucose recordings were retrospectively calibrated using capillary glucose (fingersticks) values from a self- monitored blood glucose (SMBG) meter (Contour ^®^ by Bayer), as specified by the manufacturer [18]. The SMBG values were measured four times during the study, namely before breakfast, before lunch, before dinner, and before sleeping.

At the end of the study, data from the sensor as well as SMBG values were uploaded via internet connection to CareLink iPro (https://carelink.minimed.eu/ipro/hcp/) to calibrate the CGM measurements against the capillary glucose measurements. CGM provides calibrated continuous glucose readings every 5 minutes, which were downloaded and used for analyses.

#### Processing of venous blood samples

After blood withdrawal, the serum tubes were kept at room temperature to clot and immediately centrifuged when the samples were clotted. Serum was aliquoted into 500μl tubes and promptly stored at -80°C. Glucose levels were measured using fully automated equipment with the Hitachi Modular P800 from Roche Diagnostics (Almere, the Netherlands). Coefficient of variation for measurements ranged between 0.9–3.0%.

#### Anthropometrics

At the study center, height, weight, body fat percentage, and waist and hip circumference were measured. Weight (in kilograms) was divided by the squared height (in meters) to calculate the body mass index (BMI). The percentage of body fat was measured using a bioelectrical impedance analysis (BIA) meter at a fixed frequency of 50kHz (Bodystat^®^ 1500 Ltd, Isle of Man, British Isles).

#### Calculations of measures of glycemia and glycemic variability

For the analyses, every other 5-minute CGM glucose measurement was paired with the corresponding 10-minute venous glucose measurement. Individual glucose measurements from CGM and venous blood sampling were first manually checked for unreliable measurements and technical errors and then processed as described below to derive measures of glycemia and glycemic variability.

Measures of glycemia were the 24h mean glucose level (09.00h–09.00h), and the mean glucose level during daytime (09.00h–23.00h) and nighttime (23.00h–09.00h). The analyses during daytime and nighttime were conducted to validate in more detail the CGM during the day (when participants were awake, had food intake, and capillary glucose measurement for calibration were taken) and during the night (when participants were asleep, had no food intake, and no calibration was done).

Measures of glycemic variability were of two classes, namely (i)- indices based on glucose distribution and amplitude of glucose excursions, and (ii) indices based on risk of hypo-/hyperglycemia and quality of glycemic control [19]. Indices of glycemic variability that were based on glucose distribution and amplitude of glucose excursions were the 24h standard deviation (SD), the standard deviation within series of 1 hour (SD*ws*1) and of 4 hours (SD*ws*4), the range (maximum–minimum), the interquartile range (IQR), the percentage coefficient of variation (% CV), the continuous overlapping net glycemic action over 1 hour (CONGA1) and over 4 hours (CONGA4) and the mean amplitude of glycemic excursions (MAGE). The SD*ws*1 and SD*ws*4 represent the average standard deviation of every 10-minute measurement over hourly (SD*ws*1) and four- hourly (SD*ws*4) periods of the glucose time series, and permit analysis of changes in variability by time of day [20]. CONGA1 and CONGA4 are measures for assessing intra-day variability over hourly (CONGA1) and four- hourly (CONGA4) segments of the glucose time series [21]. Except for MAGE, which was calculated using a MAGE algorithm [22] that is based on the principle of gradual (successive) approximations of glucose peaks and troughs and IQR (75th percentile–25th percentile), the other aforementioned indices were calculated using the Rodbard macro [23].

Indices based on risk of hypo-/hyperglycemia and quality of glycemic control were the J-index, hypoglycemia index and hyperglycemia index [20]. The J-index is a measure of quality of glycemic control based on mean and SD of all glucose values, and is calculated as J = 0.001 x (mean + SD)^2^. The hypoglycemia index is the weighted average of hypoglycemia values, calculated using the formula (∑ (LLTR—Glucose)^*b*^) / [N x *d*], where LLTR = Lower Limit of Target Range (we used the default value of 80mg/dL); *b* = exponent, generally in the range from 1.0 to 2.0 (we used the default value of 2.0); *d* = scaling factor to permit another form of differential weighting of hypoglycemic and hyperglycemic values (we used the default value of d = 30); and N is the total number of observations including hypo-, eu, and hyperglycemic values. The summation for hypoglycemia index was performed for only the glucose values less than 80mg/dL. The hyperglycemia index is the weighted average of hyperglycemic values, calculated using the formula (∑ (Glucose–ULTR)^*a*^) **/** [N x *c*], where ULTR = Upper Limit of Target Range (we used the default value of 140mg/dL); *a* = exponent, generally in the range from 1.0 to 2.0 (we used the default value of 1.1); *c* = scaling factor (we used the default value of c = 30); and N is the total number of observations. The summation for hyperglycemia index was performed for only the glucose values greater than 140mg/dL. The exponents *a* and *b*, and the scaling factors *c* and *d* in the formulas for hypoglycemia and hyperglycemia indices are constants that provide for differential weighting of hypo- and hyperglycemic values.

### Statistical Analysis

The accuracy of individual glucose measurements was studied by assessing the accuracy within a participant as well as for the whole study population, whereas the accuracy of the measures of glycemia and glycemic variability was assessed only for the study population.

For the analyses on accuracy of individual glucose measurements, we first calculated, per participant, a Pearson correlation coefficient between glucose measurements derived from CGM and venous blood sampling. From these individual Pearson correlation coefficients, we determined the median with the interquartile range. Secondly, we assessed the agreement between glucose measurements derived from CGM and venous blood sampling using Bland-Altman analysis [24]. The limits of agreement were studied by calculating the difference between the two methods ± 1.96 standard deviation of the difference. The Bland-Altman analyses were additionally stratified into daytime and nighttime. We additionally determined the mean absolute relative difference (MARD) of all paired points. MARD was calculated using the formula |CGM glucose—venous glucose|/venous glucose, as has been previously done in other studies [8, 11]. Furthermore, to explore the MARD values across different glucose ranges within our dataset, we calculated the mean and median Absolute Relative Differences (ARD) after dividing the glucose values into tertiles based on venous glucose values.

The comparison of level of agreement of the per-participant estimates of glycemia and glycemic variability derived from CGM and venous blood sampling were conducted with paired t-tests.

Graphs of 24hour glucose trajectories and Bland-Altman plots were drawn using GraphPad Prism version 5 (GraphPad, San Diego, CA). All statistical analyses were performed using SPSS v.20 for Windows (SPPS Inc., Chicago, IL, U.S.A.). Two-sided p-values below 0.05 were considered statistically significant.

To determine whether the results and correlations obtained were modulated by the presence of one participant with a negative Pearson correlation, sub-analyses were also conducted after excluding this participant (in N = 33).

## Results

### Characteristics of the study population

Characteristics of the study population (N = 34) are presented in Table 1. Summarily, the study population had a mean age of 65.7 years, and comprised of 44% females. Participants had a mean BMI of 25.2 kg/m^2^, and mean fasted venous glucose of 4.9 mmol/L.

**Table 1:** Characteristics of the study population.

Demographics	N = 34
Female, n (%)	15 (44.1)
Age, years	65.7 (4.8)
Weight, kg	75.5 (13.3)
BMI, kg/m2	25.2 (3.9)
Waist circumference, cm	93.1 (12.2)
Waist: hip ratio	0.90 (0.09)
Fat mass, percentage	30.8 (8.04)
Total lean mass, kg	52.3 (11.6)
Fasted (venous) glucose, mmol/L	4.9 (0.6)

Data represent mean with standard deviation unless stated otherwise.

### Accuracy of individual glucose measures obtained with CGM

**Fig 1:**
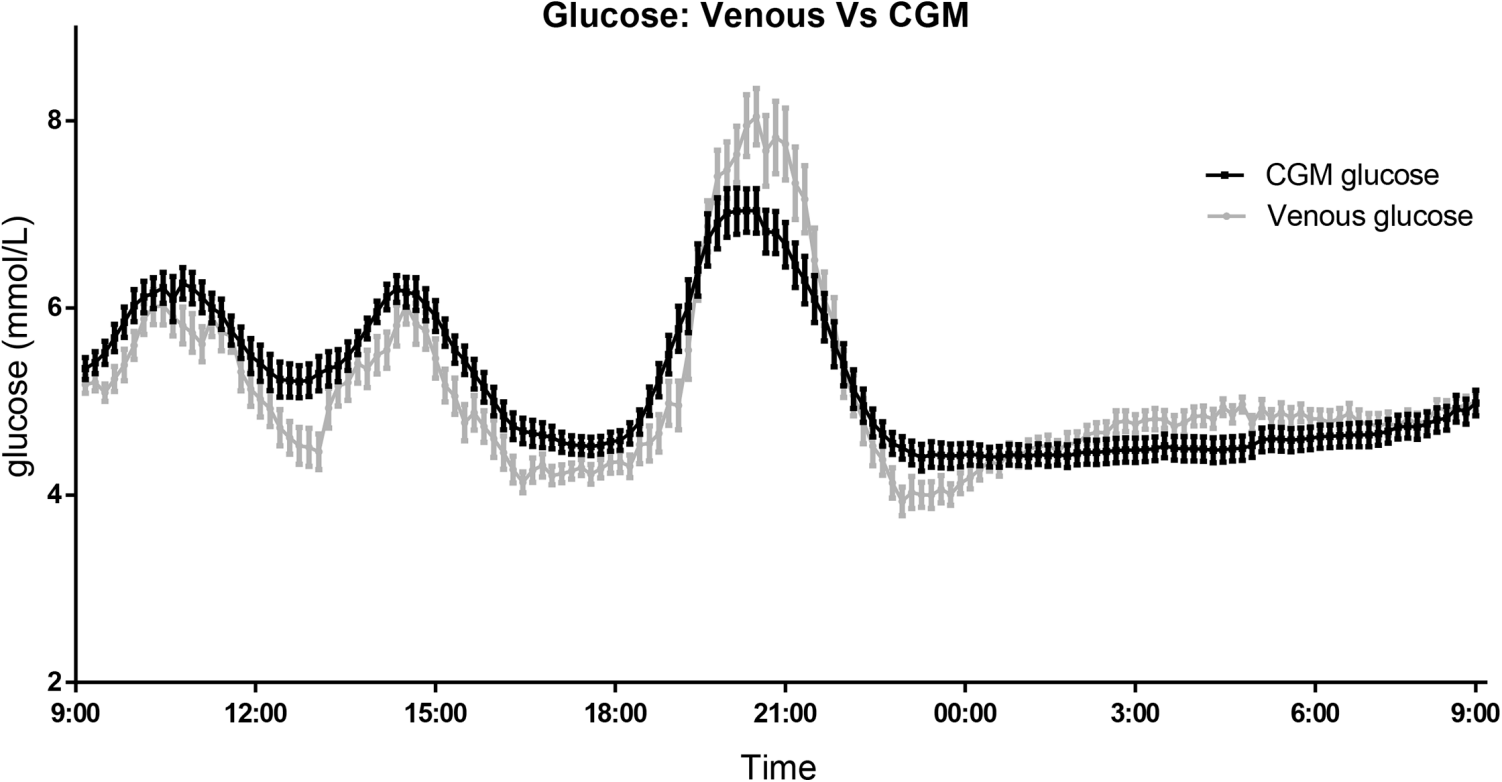
Venous- and continuous glucose monitoring (CGM)- derived glucose during 24h period. Data presented as the mean (SE) glucose level every 10 minutes. In red, the continuous glucose monitoring measurement data. In blue, the venous blood glucose measurement data.

A total of 4,523 data points derived with CGM were paired with glucose levels from simultaneously obtained venous blood samples. A graphical representation of the average glucose level (CGM and venous blood glucose) per time point is visualized in **Fig 1**.

**Fig 2:**
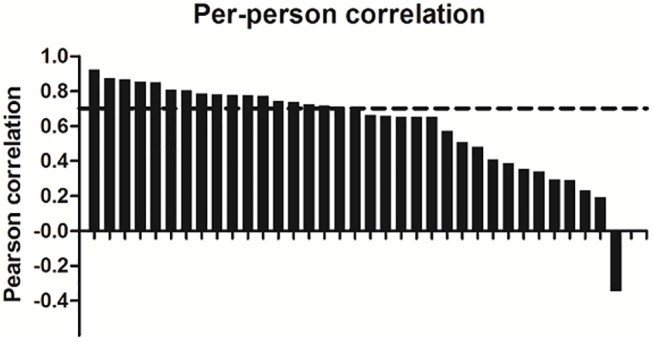
Per-person Pearson correlations coefficients between venous- and continuous glucose monitoring (CGM)- derived glucose levels. Bar chart showing the distribution of the Pearson correlations between paired CGM and venous glucose measurements determined for each of the 34 participants. Dashed line represents the median per-person Pearson correlation.

Pearson correlation coefficients between glucose measurements derived with CGM and from venous blood samples are visualized per participant in **Fig 2** (individual 24h graphs are presented in **S1 Fig**). These Pearson correlation coefficients ranged from -0.35 to 0.93 with a median Pearson correlation of 0.68 (interquartile range: 0.40–0.78).

**Fig 3:**
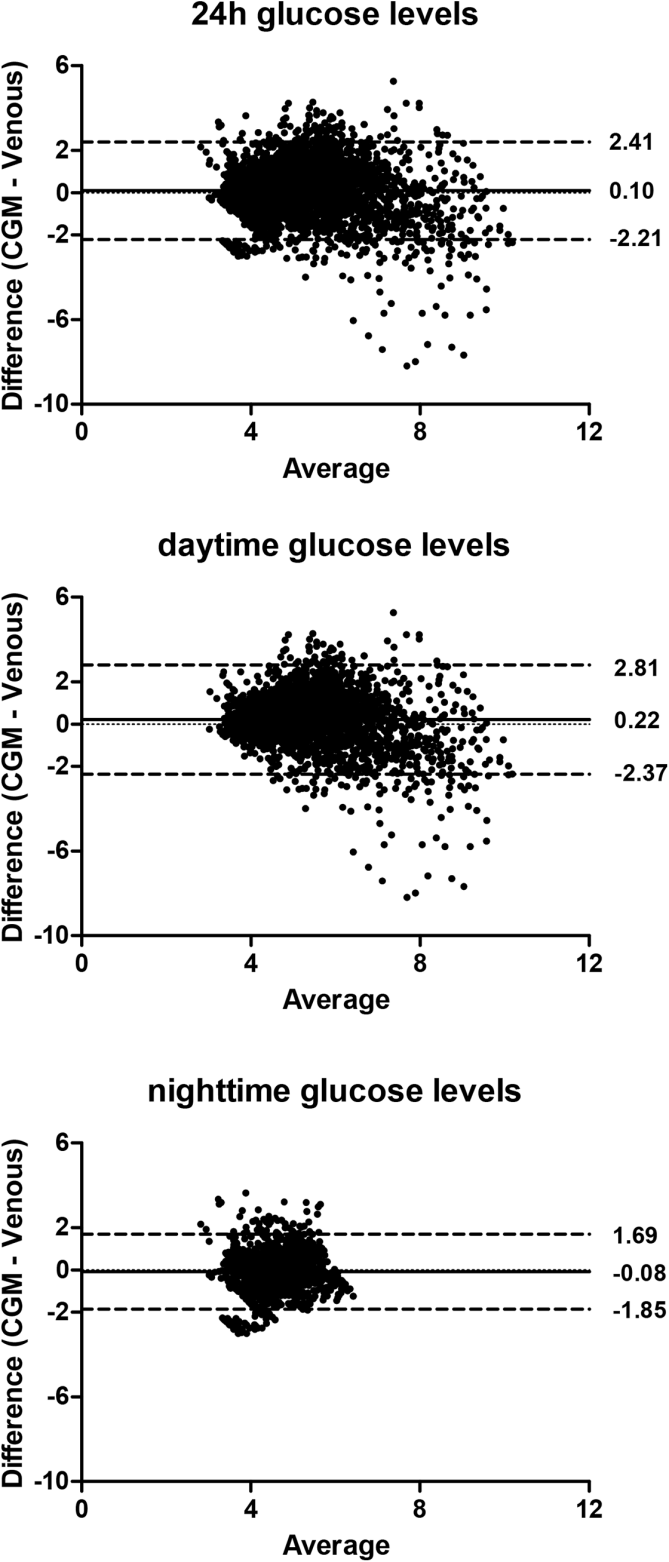
Bland-Altman plots of individual glucose measurements. Each dot represents one paired (CGM and venous) glucose measurement (N = 4,523 data points derived from 34 participants). The bias of the measurements (represented as the solid lines) and the ± 1.96 SD (dotted lines) are presented for the measurements obtained (A) over 24 hours, (B) during the day (09.00h–23.00h), and (C) during the night (23.00h–09.00h).

Bland-Altman plots of all the individual glucose measurements are presented in **Fig 3**. Compared to venous glucose measurements, glucose levels derived with CGM were on average 0.10 mmol/L higher (95% of the individual data points between -2.21 and 2.41 mmol/L) during the 24-hour period. During the day, glucose levels were 0.23 mmol/L (95% of the individual data points between -2.36–2.82 mmol/L) higher with CGM, while during the night glucose levels were 0.09 mmol/L lower with CGM (95% of the individual data points between -1.85–1.67 mmol/L). The MARD was 17.6% (SD = 17.0%) throughout the 24-hour period, 19.3% (SD = 17.1%) during the day, and 15.3% (SD = 16.5%) during the night. Next, we divided the glucose values into tertiles based on venous glucose values to explore MARD values across different glucose ranges within our dataset. The 24-hour mean (and median) Absolute Relative Difference (ARD) were 22.8 (16.99), 14.45 (11.0) and 15.65 (13.29) for tertile 1 (lowest glucose values), 2 (intermediate glucose values) and 3 (highest glucose values) respectively, as described in **S1 Table**. These results were similar after excluding the participant with a negative Pearson correlation.

### Accuracy of estimates of glycemia and glycemic variability

Agreement between calculated estimates of glycemia and glycemic variability derived from CGM and venous blood data is presented in **Table 2**. The 24-hour mean glucose level was 0.08 mmol/L (standard error [SE]: 0.08) higher with CGM than with venous blood sampling, which was not statistically significantly different (p-value = 0.35). During daytime, the mean glucose level was 0.22 mmol/L (SE: 0.09) higher with CGM than with venous blood sampling, which was significantly different (p-value = 0.02). No significant difference (p-value = 0.33) in mean nighttime glucose was observed between CGM and venous blood (difference: -0.11 mmol/L; SE: 0.12).

**Table 2:** Comparison of estimates of glycemia and glycemic variability.

	Mean CGM	Mean venous	Difference (SE)	P-value
**Glycemia**				
24h glucose	5.18 (0.46)	5.10 (0.35)	0.08 (0.08)	0.35
Daytime glucose (09.00h–23.00h)	5.61 (0.52)	5.39 (0.43)	0.22 (0.09)	0.02
Nighttime glucose (23.00h–09.00h)	4.57 (0.62)	4.68 (0.47)	-0.11 (0.12)	0.33
**Glycemic variability: glucose distributions/ excursions**				
24h standard deviation	1.07 (0.29)	1.26 (0.36)	-0.19 (0.06)	0.004
SD*ws*1	0.47 (0.09)	0.68 (0.15)	-0.21 (0.02)	<0.001
SD*ws*4	0.72 (0.18)	0.95 (0.26)	-0.23 (0.04)	<0.001
Range	4.45 (1.19)	6.24 (1.72)	-1.79 (0.25)	<0.001
IQR (median (IQR))	1.24 (1.05–1.42)	1.27 (1.00–1.46)		0.354
% Coefficient of Variation	20.9 (6.04)	24.3 (6.67)	-3.39 (1.20)	0.008
MAGE	3.02 (1.02)	3.52 (1.36)	-0.50 (0.22)	0.034
CONGA1	1.50 (0.42)	1.97 (0.61)	-0.47 (0.07)	<0.001
CONGA4	1.57 (0.51)	2.07 (0.66)	-0.50 (0.11)	<0.001
**Glycemic variability: risk and quality of glycemic control**				
J- index	0.039 (0.007)	0.041 (0.007)	0.002 (0.007)	0.341
Hypoglycemia index (median (IQR))	0.46 (0.07–2.0)	1.33 (0.35–2.15)		0.17
Hyperglycemia index (median (IQR))	0.003 (0.0–0.04)	0.031 (0.01–0.08)		<0.001

Values are mean (SD) unless otherwise indicated. Abbreviations: CGM, continuous glucose monitoring; SD, standard deviation; SE, standard error; SD*ws*1and SD*ws*4, standard deviation within time series of respectively 1 and 4 hours; CONGA1 and CONGA4, continuous overlapping net glycemic action over respectively 1 and 4 hours; and MAGE, mean amplitude of glycemic excursions.

The 24-hour standard deviation of the glucose levels derived with CGM was 1.07, whereas it was 1.26 with venous blood sampling, which was statistically significantly different (difference: -0.19 [SE: 0.06]; p-value = 0.004). Similar significant results were observed for most other measures of glycemic variability that are based on glucose distribution and amplitude of glucose excursions, namely, SDw1, SDw4, range, % CV, MAGE, CONGA1 and CONGA4, but not for IQR. On the other hand, measures of glucose variability indices that are based on risk and quality of glycemic control did not significantly differ when derived from CGM compared to venous glucose, except for the hyperglycemia index (p-value < 0.001) (**Table 2**).

Results were similar after exclusion of the participant with a negative Pearson correlation.

## Discussion

In the present study we assessed the accuracy of CGM-derived glucose levels and of CGM-derived measures of glycemia and glycemic variability in a normo-glycemic study population. The findings were three-fold. First, we observed large variation in the per-person Pearson correlation coefficients of glucose measurements derived with CGM and venous blood glucose measurements. Second, we observed no significant systematic deviation in glucose measurements derived with CGM against glucose measurements derived with venous sampling. However, variation (as assessed by the 1.96 SD intervals in the Bland-Altman plots) was large. And third, we observed that of the calculated measures of glycemia and glycemic variability, the mean glucose level during daytime was higher with CGM, and that most measures of glycemic variability were lower with CGM, especially the glucose variability measures that were based on glucose excursions.

CGM has primarily been used for self-monitoring of blood glucose in patients with diabetes [25, 26], and only recently in a number of epidemiological studies [12–14]. Although repeated venous blood sampling could be considered as the gold standard to determine glucose rhythms over the day, this technique is too invasive to be used in larger cohort studies. Also, a large number of exclusion criteria need be considered before, for example, older participants (e.g., aged 65 and above) could undergo 24-hour venous blood collection [17]. These stringent selection criteria (e.g., lack of any significant chronic disease) will result in a highly selected study population, and increase the risk of selection bias, which decreases generalizability of potential research findings. On the other hand, the CGM device can be used with less stringent selection criteria, and can be used in a home-based setting for up to a week.

The assessment of the validity of the Enlite^®^ sensor against frequently sampled venous blood has been studied before [8–11]. The MARD, which is indicative of the direction and extent of bias [11], has been previously reported to be 18.3% in subjects with diabetes over a 48-hour period [11] and 13.6% in another study over a 6-day period [8]. In line, we observed a MARD ranging from 15.25%–19.15% depending on the time of the day. After dividing the glucose values into tertiles of venous glucose, we found that MARD values were highest in the lowest glucose range, suggesting that the MARD, as a relative error, weighs more errors at lower glucose levels. In addition, studies conducted on newer generation CGMs have reported lower MARD values. For example, a comparative study reported a MARD of 17.9 for Enlite^®^ sensor (released 2010), whereas the FreeStyle Navigator (released 2012 by Abbott Diabetes Care) and G4 Platinum (released 2013 by Dexcom) had MARD values of 12.3 and 10.8 respectively. Thus, MARD values seem dependent on glucose ranges (normo-glycemic versus diabetic range), as well as on the type and generation of CGM sensor.

The accuracy of CGM-derived measures of glycemic variability has not been studied before. Currently, glycemic variability is often monitored, as diabetes complications may be associated with higher glycemic variability [26–28]. We observed that most measures of glycemic variability were lower when derived from CGM data than when derived from venous-blood data, suggesting that measures of glycemic variability were underestimated when calculated using CGM glucose values. Several probable sources of CGM underestimation have been put forth in literature [29–31]. One reason could be distortion due to blood- to- interstitium glucose kinetics, resulting in a time lag/ delay between interstitial fluid and venous blood. In our study, we used the Enlite^®^ CGM sensor which was calibrated with glucose values from a self- monitored blood glucose (SMBG) meter (Contour^®^ by Bayer). Of note, the glucometer measures capillary glucose, which is a compartment different from that from which glucose is measured both by the CGM (interstitial compartment) and by venous sampling (intravascular compartment). Thus, the existence of a physiological delay between blood glucose and interstitial glucose can hinder real- time accurate CGM glucose measurement [31].

A second possible reason could be due to inaccurate sensor calibration [29, 30], which may be affected by sample timing or level of self- monitored glucose used for calibration, or to a drift in time of sensor sensitivity. However, distortion by inaccuracy of the glucometer (Contour^®^ by Bayer) is unlikely in our case, since this device has been previously validated against a reference laboratory glucose measuring instrument [32]. According to that study, the validity of the Contour^®^ blood glucose monitoring system is above that required by the International Organization for Standardization’s International standard (ISO 15197:2003) for blood glucose monitoring systems.

Thirdly, underestimation of CGM glucose could be attributed to random zero- mean measurement noise [29]. The measurement noise component appears to decrease day after day, causing inter-day sensor variability. The measurement noise of the CGM is highest in the first day of use and decreases thereafter. Hence, the CGM sensor was inserted the day before venous sampling was initiated in our study.

We observed a large variation in accuracy between individuals, which was reflected in a wide range in per-person Pearson correlation coefficients. In one extreme case, data showed a negative correlation between CGM and venous glucose values. No technical reason was found to explain this negative correlation. Although all participants received the same meals at approximately the same time, there were differences in individual responses, as measured by CGM or venous glucose. During the day, individual glucose values were higher when measured in the interstitial fluid using CGM than in serum (notably 0.23 mmol/L). A similar difference was observed when we calculated the mean glucose level during daytime. However, the variation in the per-person Pearson correlations is not unexpected, as individual differences may exist in how well the CGM calibration algorithm “fits” individual physiology [4]. Other reasons for the high variation in per-person Pearson correlation coefficients could include tissue reactions to the implanted sensors (e.g., inflammation, fibrosis, and vessel regression) [33]. Also, the implanted glucose sensor could have been placed close to a blood vessel, which has been previously associated with extended (average 7–15 minute) delay in interchange between interstitial fluid and venous blood [33]. These factors could contribute to a larger discordance in CGM and venous glucose values when matched based on time points. Nevertheless, despite the inclusion of one participant with a negative correlation in the analysis, our results (e.g. MARD, median Pearson correlation) are comparable to previously published studies [8, 11]. Furthermore, across the whole study population, we observed good agreement between individual glucose levels measured in serum and in interstitial fluid in normo-glycemic participants.

Compared to daytime venous glucose, we observed a higher mean CGM glucose during the day. This should be taken into account when the purpose of a study involves a cut-off determined on the basis of CGM data, as this could influence the results- the higher daytime glucose could result in a number of false-positives. Moreover, the higher standard deviation that we observed could affect the statistical power of a study. A consequence of a higher standard deviation with CGM is that CGM studies would need to be conducted with larger sample sizes than studies with venous blood sampling (**S2 Fig**). For example, when the expected differences between two groups is 0.25 mmol/L in 24-hour mean glucose level, a study using venous blood sampling would comprise 31 participants in each group, whereas a study using CGM would comprise 53 participants in each group.

A limitation of our study is that it has a limited sample size (N = 34), which is somewhat smaller than the other conducted validation studies in this field [8–11]. Another potential limitation is that venous glucose was measured in serum samples. However, the samples were centrifuged immediately after clotting, thus preventing glycolysis. The main strength is that the data with both sampling methods comprise glucose levels collected over a 24-hour period. This way, the validity of the sampling method could be studied in more detail. Furthermore, within the 24-hour study period, environment, physical activity, sleeping and feeding conditions were standardized. The study population was therefore more homogenous.

In conclusion, there is good agreement between individual glucose measurements derived with CGM and venous blood. However, the accuracy of measures of glycemia and most measures of glycemic variability deviated significantly, a fact that needs to be taken into account in future studies using CGM.

## Supporting Information

S1 Fig Per- person graphs of 24-hour glucose rhythms. Each dot represents a CGM glucose (red) or venous glucose (blue) measurement per 10-minute time point over a period of 24hours.
(DOCX)

S2 Fig Sample size calculations. Figure depicts the sample sizes required to observe a statistically significantly difference between two study groups (alpha = 0.05; power = 0.8). The vertical dashed line depicts a hypothetical expected difference between two study groups. The horizontal dashed lines depict the number of participants required in both study groups to observe this difference. Dotted curved line represents CGM whereas the solid curved line represents venous blood sampling.
(TIF)

S1 Table Mean and median absolute relative difference in tertiles of venous glucose. Abbreviations: ARD, absolute relative difference; SD, standard deviation, IQR, interquartile range.
(DOCX)

## Abbreviations

ARD: Absolute relative difference
CGM: Continuous glucose monitor
CONGA: Continuous overlapping net glycemic action
CONGA1: Continuous overlapping net glycemic action over one hour
CONGA4: Continuous overlapping net glycemic action over four hours
CV: Coefficient of variation
IQR: Interquartile range
LLS: Leiden longevity study
MAGE: Mean amplitude of glycemic excursions
MARD: Mean absolute relative difference
SE: Standard error
SD: Standard deviation
SD*ws1*: Standard deviation within time series of one hour
SD*ws4*: Standard deviation within time series of four hours
SMBG: Self-monitoring of blood glucose
