# Supplementary material for: Accuracy of Continuous Glucose Monitoring Measurements in Normo-Glycemic Individuals
Source: PLoS One. 2015 Oct 7;10(10):e0139973. doi: 10.1371/journal.pone.0139973 (PMC4596806; doi:10.1371/journal.pone.0139973)
Supplement: S1 Fig — Each dot represents a CGM glucose (red) or venous glucose (blue) measurement per 10-minute time point over a period of 24hours. (DOCX) [file pone.0139973.s001.docx]

**Supplementary figure S1. Per- person graphs of 24-hour glucose rhythms.**

Each dot represents a CGM glucose (red) or venous glucose (blue) measurement per 10-minute time point over a period of 24hours.


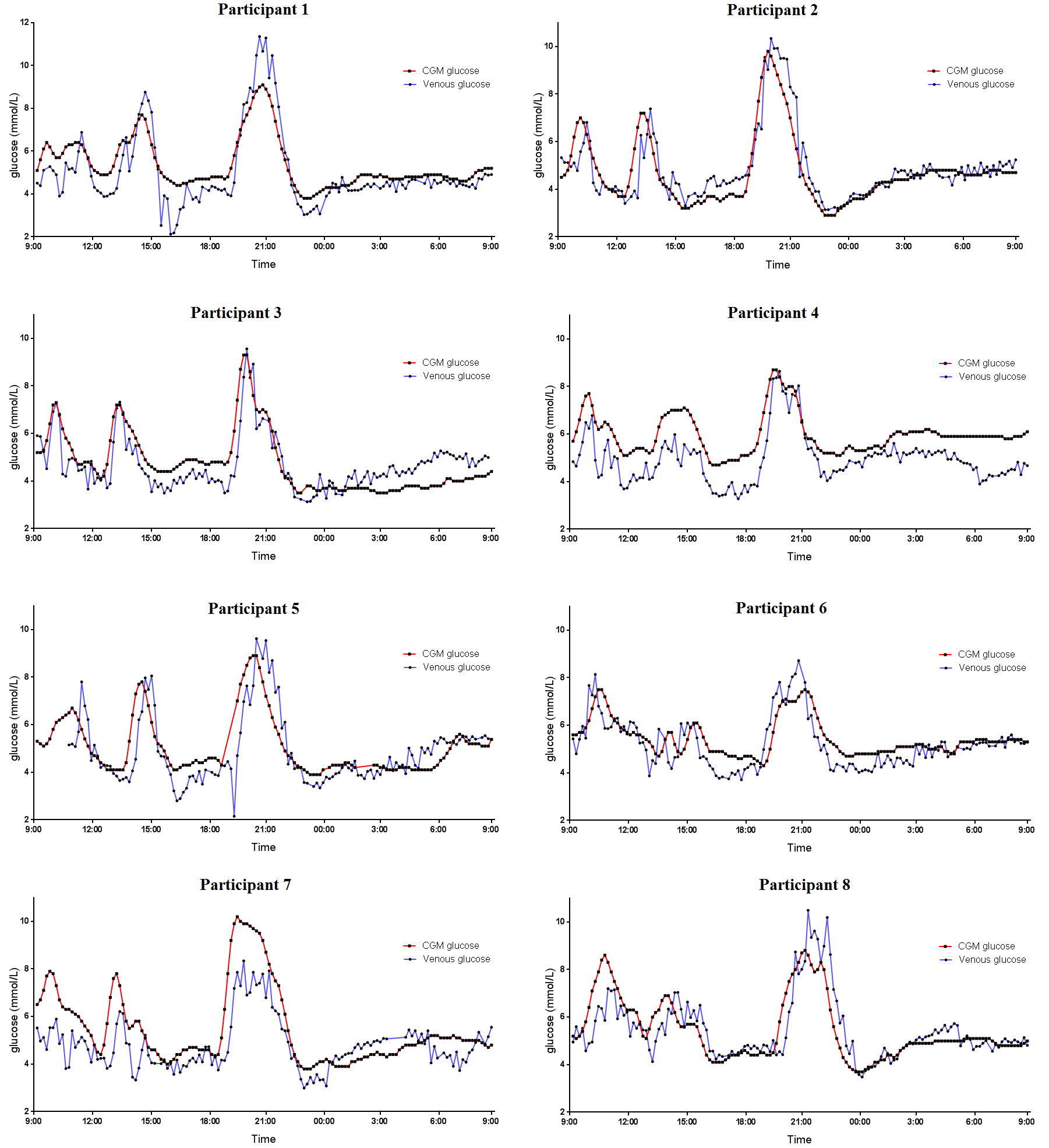


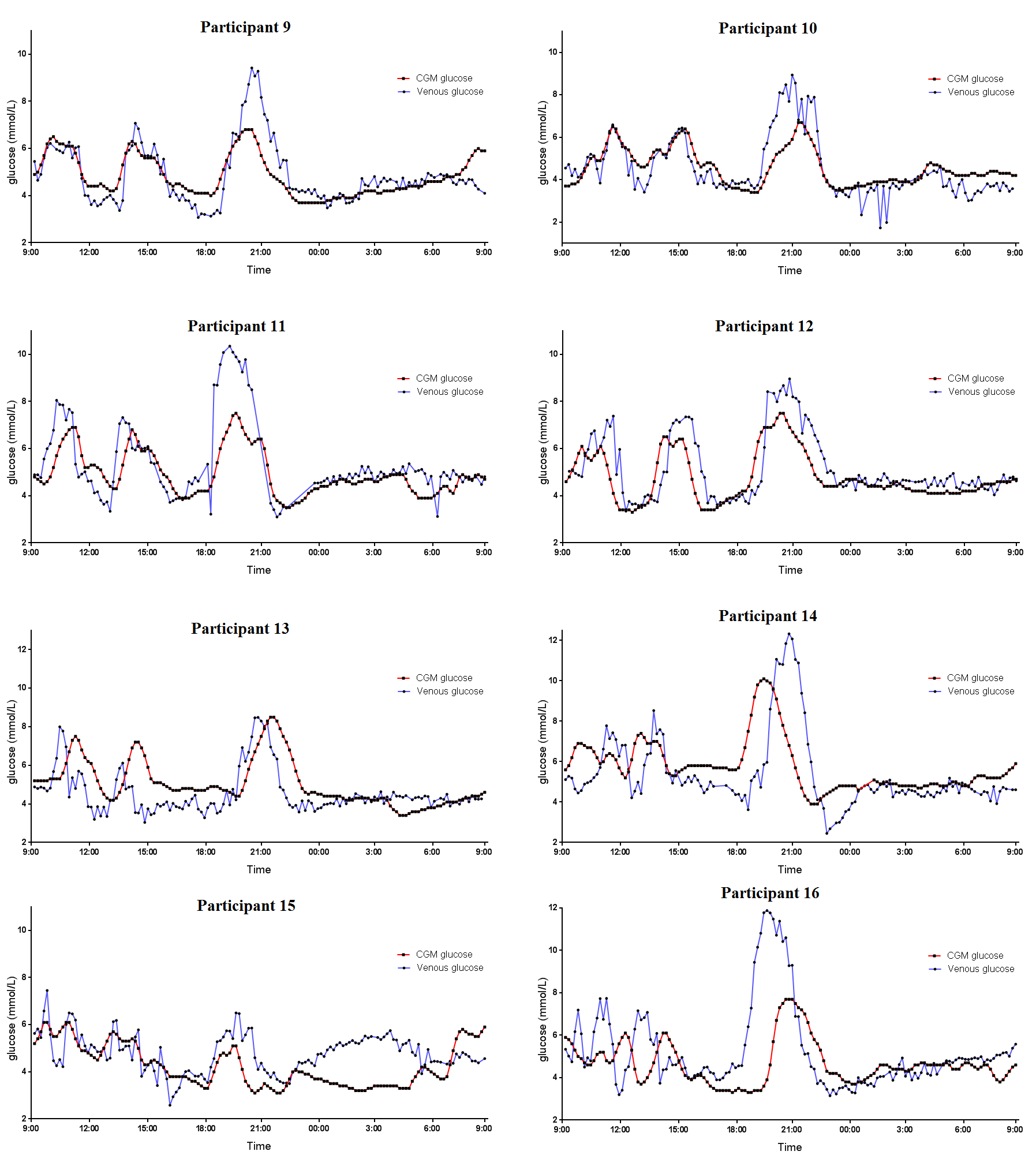


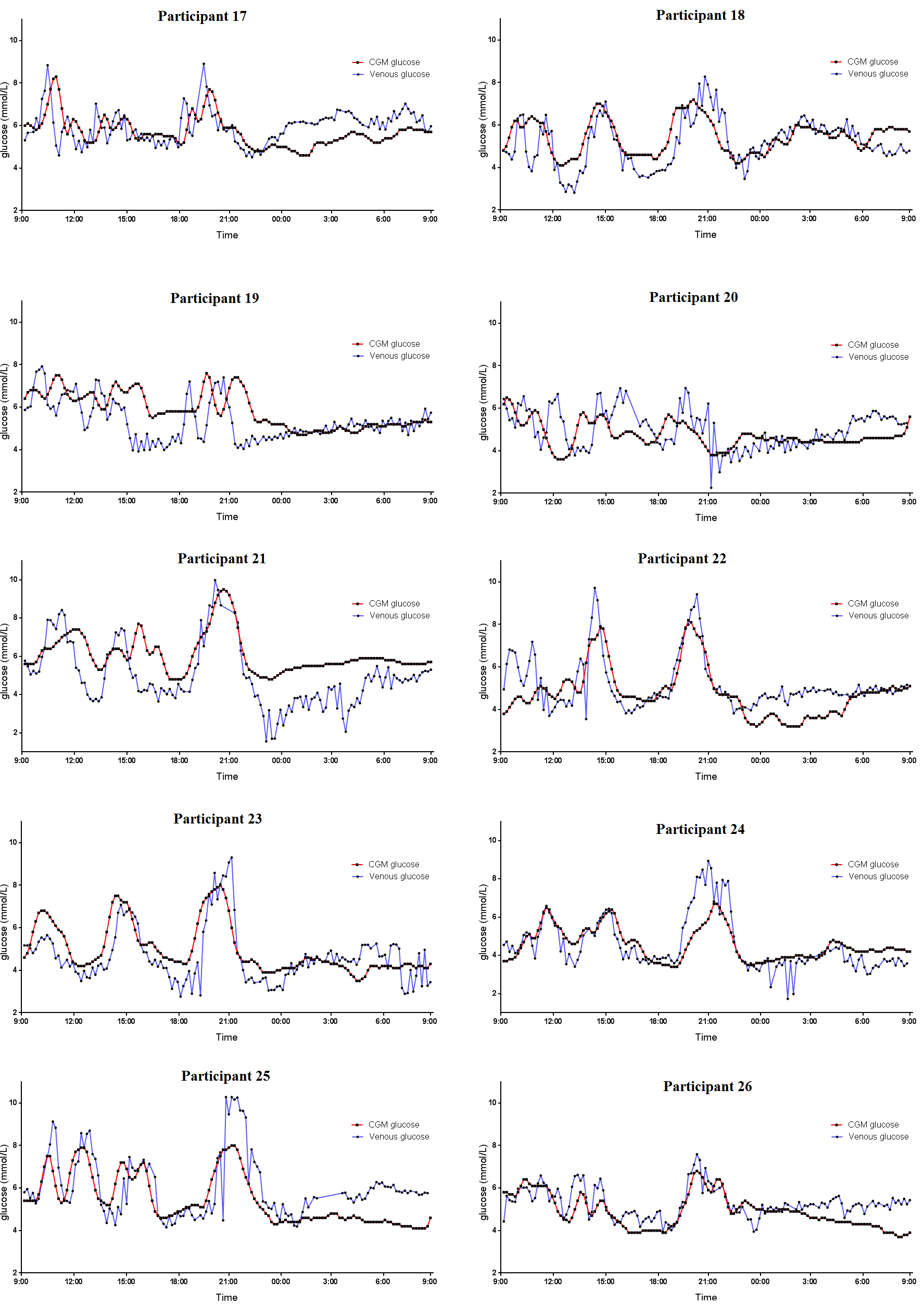


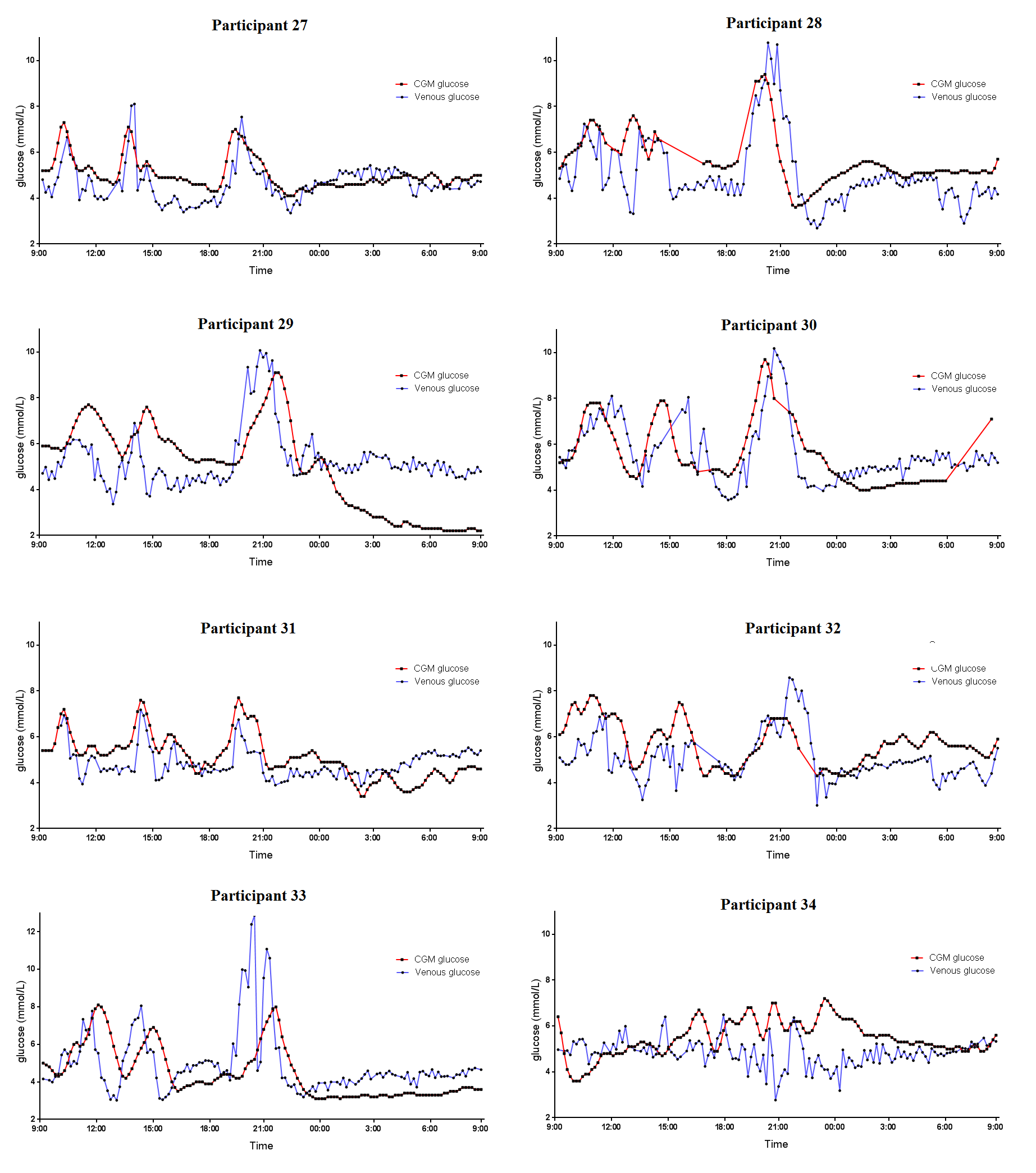


**Supplementary Figure 2: Sample size calculations**


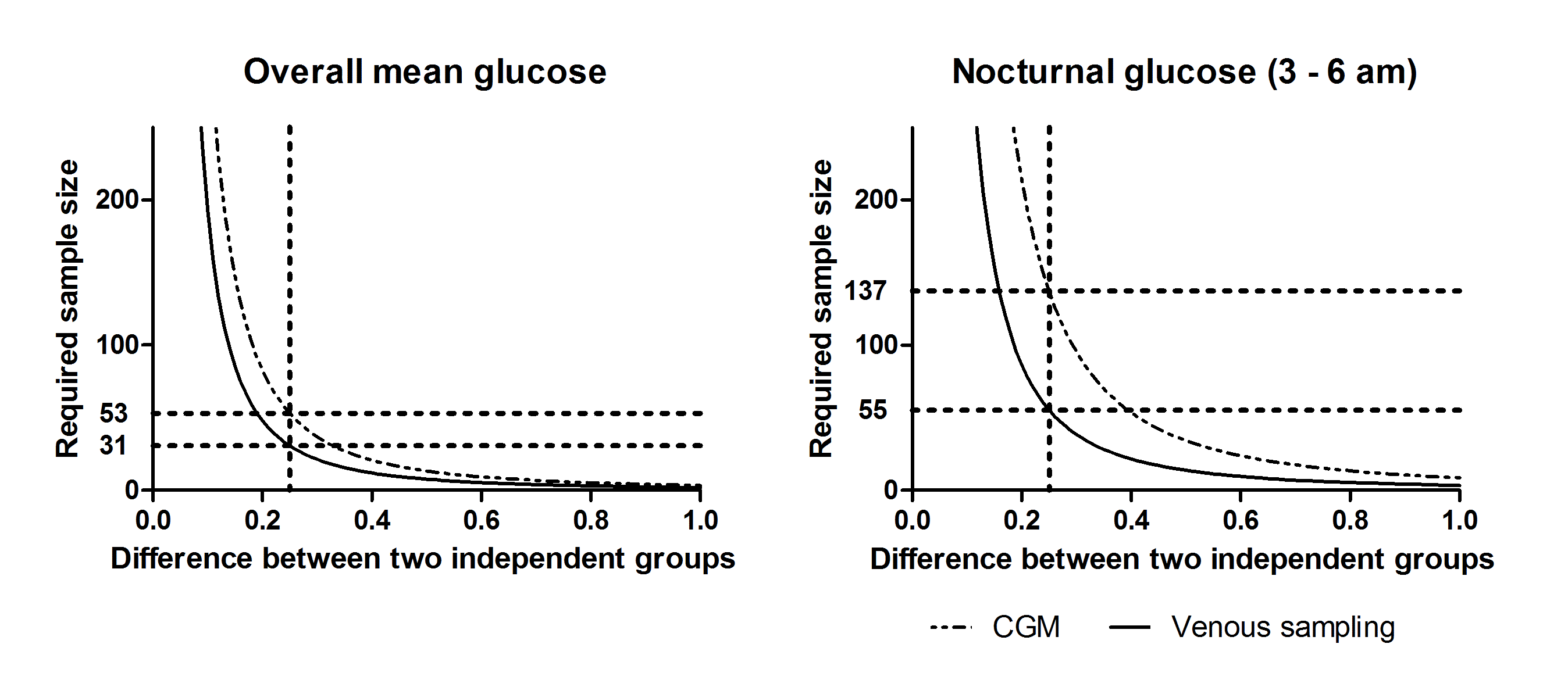


Figure depicts the sample sizes required to observe a statistically significantly difference between two study groups (alpha = 0.05; power = 0.8). The vertical dashed line depicts a hypothetical expected difference between two study groups. The horizontal dashed lines depict the number of participants required in both study groups to observe this difference. Dotted curved line represents CGM whereas the solid curved line represents venous blood sampling.
