# Supplementary material for: Accuracy of Continuous Glucose Monitoring Measurements in Normo-Glycemic Individuals
Source: PLoS One. 2015 Oct 7;10(10):e0139973. doi: 10.1371/journal.pone.0139973 (PMC4596806; doi:10.1371/journal.pone.0139973)
Supplement: S1 Table — Abbreviations: ARD, absolute relative difference; SD, standard deviation, IQR, interquartile range. (DOCX) [file pone.0139973.s003.docx]

**Supplementary Table 1: Mean and median absolute relative difference in tertiles of venous glucose**

| Supplementary Table 1: Mean and median absolute relative difference in tertiles of venous glucose | | | | | |
| --- | --- | --- | --- | --- | --- |
|  | | **Tertile 1**  (1.55-4.45 mmol/L) | **Tertile 2**  (4.46-5.23 mmol/L) | **Tertile 3** (5.24-12.88 mmol/L) | **All**  (1.55-12.88 mmol/L) |
| 24h glucose | Mean ARD (SD) | 22.80 (22.6) | 14.45 (12.9) | 15.65 (12.1) | 17.64 (16.9) |
|  | Median ARD (IQR) | 16.99  (7.9, 29.8) | 11.00  (4.9, 20.2) | 13.29  6.41, 22.1) | 13.45  (6.2, 23.6) |
| Daytime glucose (09.00h – 23.00h) | Mean ARD (SD) | 25.51(22.1) | 17.43 (14.4) | 15.67 (12.3) | 19.25 (17.1) |
|  | Median ARD (IQR) | 20.27  (10.2, 33.5) | 14.05  (6.71, 24.4) | 13.11  (6.25, 22.6) | 15.29  (7.3, 26.1) |
| Nighttime glucose (23.00h - 09.00h) | Mean ARD (SD) | 19.20 (22.8) | 12.32 (11.1) | 15.55 (11.2) | 15.29 (16.5) |
|  | Median ARD (IQR) | 13.11  (5.67, 23.7) | 9.11  (4.0, 17.7) | 14.23  (6.9, 20.9) | 11.11  (5.0, 20.3) |

Abbreviations: ARD, absolute relative difference; SD, standard deviation, IQR, interquartile range
